# 

**Published:** 1891-05-09

**Authors:** 


No. 241, Vol. X.— May 9th, 1891.

[Registered at the General Post Office as a Newspaper for transmission in the United Kingdom and Abroad.]

Per Annum, 6s. 6d.; post free.

Monthly Parts, 6d.; post free 8d.

Ditto per Annum, 8s., post free.

### THE HOSPITAL

GLASGOW WESTERN INFIRMARY — ST THOMAS'S HOSPITAL — NORFOLK AND NORWICH HOSPITAL

A WEEKLY JOURNAL OF

Science, Medicine, Nursing and Philanthropy.

SATURDAY, MAY 9th, 1891.

**Oxford on its Mettle** . . . . . . 61

**Passing Topics.**—Charity Dinners . . . . 62

**The Doctor's Armchair.**—"Natura non Facit Saltum" . 63

**The Microscope in Medicine**—By FRANK J. WETHERED, M.D., XIV.—The Examination of Vomit, Fæces, and Morbid Fluids . . . . . 64

**Everybody's Page** . . . . . . 65

**Notes and News** . . . . . . 66

**Annotations.**—Dr. Steele on Medical Charities—The Dulness of Domestic Service—Eight Hours a Day . . . 68

**General Charities** . . . . . . 67

**The Practitioner's Mirror and Retrospect:**—

MEDICINE.—The Cholera . . . . . 69

SURGERY.—Ingleby Lectures: Lecture V. . . . 70

THERAPEUTICS.—Euphrasia Officinalis . . . 71

**Scraps and Gleanings** . . . . . 71

**The Lords' Committee** . . . . . 72

**Sale of Professor Koch's Lymph.—Books Received** 72

**The Student of Medicine.**—Appointments—Vacancies—Scholarships and Prizes—Pass Lists.

### EXTRA SUPPLEMENT—THE NURSING MIRROR.

**En Passant.**—South London Association—Short Items—Hedda Gabler—Royal Berkshire Hospital—Leicester Institution . . . . . xxxi

**Lectures on Surgical Ward Work and Nursing.**—By ALEX. MILES, M.B. Edin., C.M., F.R.C.S.E.—Lecture XXIII.—Plaster of Paris Case . . . xxxii

**Examination Questions** . . . . . xxxii

**Nursing Medals and Certificates.**—The Khedive's Bronze Star . . . . . xxxiii

**Notes and Queries.—Death in Our Ranks** . . xxxiii

**For Reading to the Sick.**—Forgiveness . . . xxxiii

**A Letter from Kansas** . . . . . xxxiv

**Private Nursing** . . . . . xxxiv

**Amusements** . . . . . xxxv

**Appointments** . . . . . xxxv

**Presentations** . . . . . xxxvi

**How to Rest** . . . . . xxxvi

**Amusements and Relaxation** . . . . xxxvi

